# Pirfenidone Inhibits Cell Proliferation and Collagen I Production of Primary Human Intestinal Fibroblasts

**DOI:** 10.3390/cells9030775

**Published:** 2020-03-22

**Authors:** Yingying Cui, Mengfan Zhang, Changsen Leng, Tjasso Blokzijl, Bernadien H. Jansen, Gerard Dijkstra, Klaas Nico Faber

**Affiliations:** 1 Department of Gastroenterology and Hepatology, University Medical Center Groningen, University of Groningen, 9713 GZ Groningen, The Netherlands; y.cui@umcg.nl (Y.C.); m.zhang@umcg.nl (M.Z.); t.blokzijl@umcg.nl (T.B.); b.h.jansen@umcg.nl (B.H.J.); gerard.dijkstra@umcg.nl (G.D.); 2 Department of Biomedical Sciences of Cells and Systems, section Molecular Cell Biology, University Medical Center Groningen, University of Groningen, 9713 GZ Groningen, The Netherlands; c.leng@umcg.nl; 3 Department of Laboratory Medicine, University Medical Center Groningen, University of Groningen, 9713 GZ Groningen, The Netherlands

**Keywords:** intestinal fibrosis, pirfenidone, mTOR, TGF-β1, collagen, fibroblast, inflammatory bowel disease

## Abstract

Intestinal fibrosis is a common complication of inflammatory bowel disease. So far, there is no safe and effective drug for intestinal fibrosis. Pirfenidone is an anti-fibrotic compound available for the treatment of idiopathic pulmonary fibrosis. Here, we explored the anti-proliferative and anti-fibrotic properties of pirfenidone on primary human intestinal fibroblasts (p-hIFs). p-hIFs were cultured in the absence and presence of pirfenidone. Cell proliferation was measured by a real-time cell analyzer (xCELLigence) and BrdU incorporation. Cell motility was monitored by live cell imaging. Cytotoxicity and cell viability were analyzed by Sytox green, Caspase-3 and Water Soluble Tetrazolium Salt-1 (WST-1) assays. Gene expression of fibrosis markers was determined by quantitative reverse transcription PCR (RT-qPCR). The mammalian target of rapamycin (mTOR) signaling was analyzed by Western blotting and type I collagen protein expression additionally by immunofluorescence microscopy. Pirfenidone dose-dependently inhibited p-hIF proliferation and motility, without inducing cell death. Pirfenidone suppressed mRNA levels of genes that contribute to extracellular matrix production, as well as basal and TGF-β1-induced collagen I protein production, which was associated with inhibition of the rapamycin-sensitive mTOR/p70S6K pathway in p-hIFs. Thus, pirfenidone inhibits the proliferation of intestinal fibroblasts and suppresses collagen I production through the TGF-β1/mTOR/p70S6K signaling pathway, which might be a novel and safe anti-fibrotic strategy to treat intestinal fibrosis.

## 1. Introduction

Inflammatory bowel diseases (IBD), e.g., Crohn’s Disease and ulcerative colitis, are complex diseases, characterized by chronic and recurrent inflammation in the intestine [1]. Typically diagnosed in early adulthood, patients require life-long disease management without a curative therapy available at this moment. The prevalence of IBD has increased significantly in the past decades and is expected to increase even further, especially in countries adopting a Western lifestyle [2]. Intestinal fibrosis is a severe and common complication of IBD and is increasingly recognized as a therapeutic problem [3]. Chronic inflammation leads to damage to the intestinal tissue. In response, intestinal myofibroblasts become activated and secrete extracellular matrix (ECM) such as collagen and fibronectins under the regulation of TGF-β1 and several other pro-fibrotic and anti-fibrotic factors. Intestinal fibrosis develops when the ECM production exceeds the co-induced ECM degradation and may lead to organ stenosis and malfunction [3,4]. Despite the introduction of novel therapeutics for IBD in the past two decades, the incidence of fibrosis-induced intestinal strictures has not significantly changed in these patients [5]. Up to 30–60% of Crohn’s disease patients experience intestinal stenosis and bowel obstruction. Short strictures can be dilated by endoscopic balloon dilatation, but approximately 75% of subjects need redilatation and 30–40% require surgical resection [5,6,7,8]. While this improves quality of life directly after surgery, stenotic bowel obstruction is bound to recur in approximately 50% of the patients within 20 years [9]. Thus, there is an urgent need to elucidate the pathophysiological mechanisms of intestinal fibrosis at the cellular and molecular level in order to develop safe and effective drugs to prevent and treat intestinal fibrosis. TGF-β1 has been identified as an important factor during fibrogenesis as it promotes ECM protein synthesis and inhibits ECM degradation [10]. Also, TGF-β1 is known to activate mTORC1 signaling pathway, which is a central regulator of cell metabolism, proliferation, and protein synthesis [11,12]. Important downstream execution proteins are 4E-BP1, a translation repressor protein, and p70S6K that targets the S6 ribosomal protein [13,14].

Pirfenidone is a pyridone compound with anti-fibrotic properties in idiopathic pulmonary fibrosis (IPF), liver cirrhosis, and cardiac fibrosis [15,16,17]. Moreover, pirfenidone significantly suppressed TGF-β1-induced ECM synthesis in a mouse model with renal fibrosis [18]. In a phase III randomized, double-blind, placebo-controlled, and multinational clinical trial (ASCEND), pirfenidone significantly improved progression-free survival and reduced the number of IPF patients who had a decline in forced vital capacity [17].

In this study, we investigated the effect of pirfenidone on primary human intestinal fibroblasts (p-hIFs), including its effect on TGF-β1-mediated mTOR-p70S6K1 signaling.

## 2. Materials and Methods

### 2.1. p-hIFs Isolation and Culture

The procurement of a part of the colon for research was approved by the National Discussion of the Procurement Teams (Landelijk OveRleg Uitname Teams) from the Dutch Transplantation Association (Nederlandse Transplantatie Vereniging) and performed after written informed consent from the relatives. All the procedures were performed according to Helsinki Declaration. Primary human colon fibroblasts were isolated and cultured as previously described [19]. Fresh transplantation surgical specimens from morphological normal ascending colon tissue were obtained from the donor. Colon tissue was cut into small pieces and placed in T25 cell culture flasks with culture medium: Dulbecco’s Modified Eagle Medium containing 20% heat-inactivated fetal calf serum, 1X MEM Non-Essential Amino Acid, 100 µg/mL gentamycin, 200 u/mL of penicillin, 200 µg/mL of streptomycin, and 2.5 µg/mL of Amphotericin B (all Gibco™ by Life technologies, Bleiswijk, The Netherlands) in a humidified incubator at 37 °C and 5% CO_2_. After p-hIFs grew from the tissue, the tissue was removed from the flask. The confluency of the p-hIFs reached up to 70–80% after approximately 3–4 weeks. All experiments were performed with at least 3 independent p-hIF isolates.

### 2.2. Proliferation Assays

#### 2.2.1. Real-Time Cell Analysis (RCTA)

Experiments of p-hIF proliferation were performed using the xCELLigence Real-Time Cell Analysis (RTCA, xCELLigence RTCA DP, ACEA Biosciences Inc., San Diego, CA, USA) as previous described [20]. p-hIFs were seeded in an E-16 plate with a density of 2500 cells/well in 200 µL culture volume. E-16 plates carry sensor microelectrodes to measure electronic impedance that represents the cell confluency. The impedance of electron flow caused by adherent cells is reported using a unitless parameter called Cell Index (CI), where CI = (impedance at time point *n*—impedance in the absence of cells)/nominal impedance value. The impedance was recorded at 15 min intervals to continuously monitor the proliferation of p-hIFs. p-hIFs were refreshed with new medium and treated with different concentrations of pirfenidone after 18 h. Results were analyzed by RTCA Software (v1.2, ACEA Biosciences Inc.).

#### 2.2.2. BrdU Assay

The BrdU assay was performed according to the standard protocol of the manufacturer (Roche, Mannheim, Germany). p-hIFs were seeded in a 96-well plate overnight and were treated with pirfenidone (0, 0.5, 1, 2 mg/mL) for 72 h. BrdU (10 μmol/L) was added and incubated in the final 24 h. After fixation, p-hIFs were incubated with 100 µL/well anti-BrdU-POD working solution for 90 min at room temperature. After 15 min of incubation with the substrate solution, the proliferation measurement was conducted using the BrdU incorporation ELISA kit (all from Roche, Mannheim, Germany).

#### 2.2.3. p-hIFs Cell Counting

p-hIFs were seeded in 6-well plates with a density of 8 × 10^5^ cells/well. After exposure to various concentrations of pirfenidone for 72 h, wells were washed with PBS and p-hIFs were dissociated using trypsin. Then p-hIFs were centrifuged and resuspended in a small volume of culture medium. Numbers of p-hIFs were quantified using a TC20 cell counter (Bio-Rad Laboratories, Inc., Hercules, CA, USA).

### 2.3. Real-Time Imaging of Cell Motility

p-hIFs were seeded in a 8-well chamber plate (Lab-Tek II, 155409, Thermo Scientific, Waltham, MA, USA) in 250 µL medium at a density of 1000 cells/well and cultured overnight, after which they were exposed to 0, 1, and 2 mg/mL pirfenidone for 24 h. Next, the plate containing the p-hIFs was transferred to a live cell imaging platform in a DeltaVision microscope (GE Healthcare Bio-sciences, Marlborough, MA, USA) and p-hIF motility was monitored for 15 h. Images were taken every 5 min using a 40 × oil objective with DIC channels and cell motility was analyzed with Imaris software (v8.0, Oxford Instruments, Zurich, Switzerland; https://imaris.oxinst.com/downloads).

### 2.4. Cytotoxicity and Cell Viability Assays

#### 2.4.1. Sytox Green Assay

p-hIFs were seeded in the 96-well plate with a density of 2500 cells/well. p-hIFs were exposed to pirfenidone for 72 h. p-hIFs exposed to H_2_O_2_ (5 mmol/L) were included as positive (necrotic) control group [21]. Sytox Green (10 µM; Life Technologies) nucleic acid stain was added to tested wells for 15 min, followed by fluorescence microscopy using a Leica DMI6000 microscope (Leica Microsystems GmbH, Wetzlar, Germany).

#### 2.4.2. Caspase-3 Assay

Caspase-3 activity (as marker for apoptosis) was quantified as described earlier [22]. After 72 h of treatment with pirfenidone, p-hIFs were scraped and cell lysates were obtained after three times of freezing (−80 °C) and thawing (37 °C) followed by centrifugation for 5 min at 12,000× *g*. 20 µg protein per sample was used to quantify caspase-3 activity.

#### 2.4.3. WST-1 Assay

The p-hIF cell viability assay was quantified using the Cell Proliferation Reagent Water Soluble Tetrazolium Salt-1 (WST-1, 11644807001 Roche) according to the manufacturer’s protocol. p-hIFs were seeded in a 12-well plate with a density of 4 × 10^4^ cells/well and were exposed to pirfenidone for 72 h. Thus, 10 µL of WST-1 solution per 100 µL medium was added to the growing p-hIFs and was next incubated at 37 °C for 90 min after which the WST-1-containing medium was transferred to a new 96-well plate for analysis.

### 2.5. Quantitative Real–Time PCR (RT-qPCR)

p-hIFs were seeded in 6-well plates with a density of 8 × 10^5^ cells/well and were exposed to pirfenidone with or without TGF-β1 for 72 h. Total mRNA was extracted from scraped p-hIFs using TRIzol reagent (Sigma-Aldrich, Zwijndrecht, The Netherlands). RNA concentrations were determined with a NanoDrop 1000 spectrophotometer (Thermo Fisher Scientific, Wilmington, DE, USA). RT-qPCR was performed using 7900HT fast Real-Time PCR system (Applied Biosystems, Bleiswijk, The Netherlands) as previously described [23]. The TaqMan primers and probes and SYBR green primers used are shown in Supplementary Tables S1 and S2. *18S* was used to normalize the mRNA level.

### 2.6. Immunofluorescence Microscopy (IF)

p-hIFs were seeded in 12-well plates (4 × 10^5^ cells/well) containing coverslips. After 72 h of different treatments, coverslips were rinsed with PBS, fixed with 4% paraformaldehyde for 10 min, and permeabilized with 0.1% Triton X-100 for 10 min at room temperature. Non-specific antibody binding was blocked with 3% bovine serum albumin/PBS solution for 30 min. Then, coverslips were incubated with primary collagen I antibody (1:1000, 1310-01, Southern Biotech, Birminghan, UK) for 1 h at 37 °C. Afterward, coverslips were rinsed with PBS three times and incubated with Alexa-Fluor488-conjugated rabbit anti-goat secondary antibodies (1:400 A11008; Molecular Probes, Leiden, The Netherlands) for 30 min. Nuclei were stained with Mounting Medium with 4’,6-diamidino-2-phenylindole (DAPI H-1200 Vector Laboratories, Peterborough, UK). Images were taken using a Leica DMI6000 fluorescence microscope (Leica Microsystems GmbH).

### 2.7. Western Blotting

p-hIFs were lysed with cell lysis buffer containing 25 mM HEPES, 150 mM KAc, 2 mM EDTA, and 0.1% NP-40 (all from Sigma-Aldrich) supplemented with protease inhibitors on ice. Protein concentrations were quantified using the Bio-Rad protein assay (Bio-Rad). Equal quantities of protein were separated by 5–12% gradient sodium dodecyl sulfate polyacrylamide gel electrophoresis. Proteins were transferred to membranes with the Trans-Blot Turbo transfer system (Bio-Rad). After 1 h of blocking using 2% bovine serum albumin/PBS-Tween, membranes were incubated with the primary antibody (antibodies catalog numbers and dilutions supplied in Supplementary Table S3) at 4 °C overnight. Then membranes were washed with three times of PBS-Tween and incubated with horseradish-peroxidase conjugated secondary antibody for 1 h. Glyceraldehyde 3-phosphate dehydrogenase (GAPDH) was used as the reference protein. The signals were detected by chemidoc XRS system and Image Lab ver3.0 (Bio-Rad).

### 2.8. Statistical Analysis

Statistical analyses were performed with Graphpad Prism 7 (Graphpad Software, San Diego, CA, USA). All data presented as mean ± SEM. Statistical differences between two groups were analyzed by using unpaired *t*-test. If more than two groups were evaluated, the groups were analyzed by using one-way ANOVA with Dunnett or Turkey test. A *p*-value < 0.05 was considered as statistically significant.

## 3. Results

### 3.1. Pirfenidone Suppresses the Proliferation of p-hIFs, Which is Reversible

**Figure 1:**
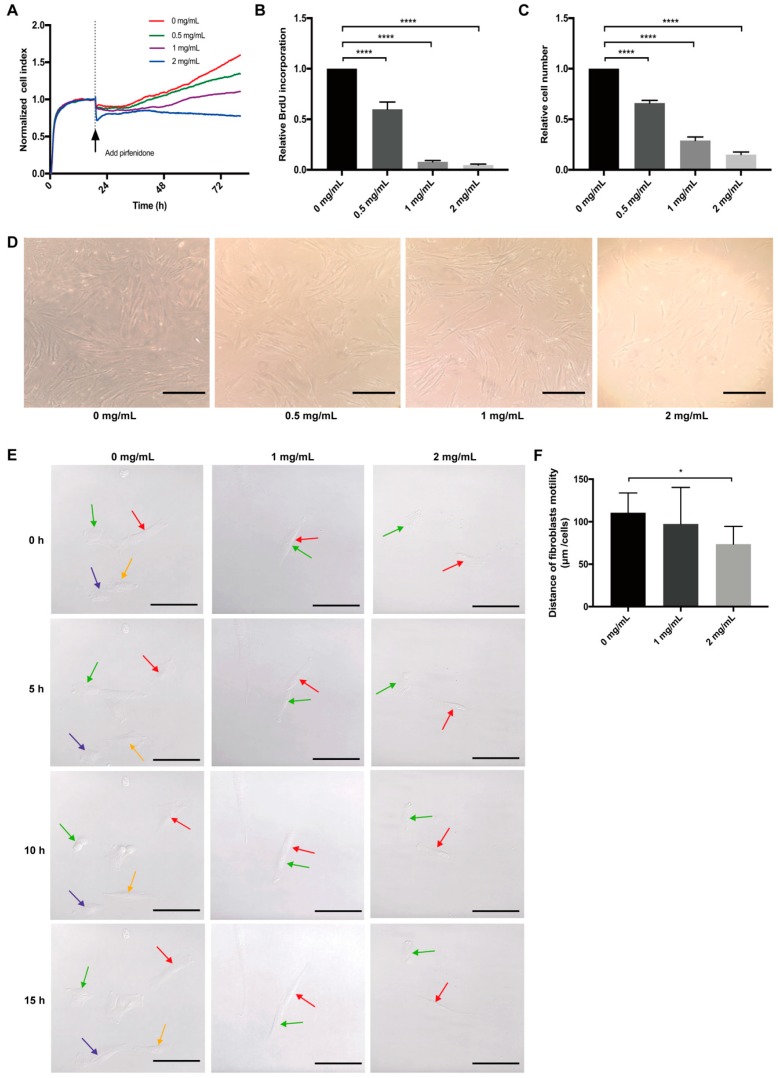
Pirfenidone suppresses the proliferation of primary human intestinal fibroblasts (p-hIFs). (**A**) Intestinal fibroblasts were seeded in the xCELLigence system for 18 h and were then exposed to increasing concentrations of pirfenidone (0, 0.5, 1, and 2 mg/mL) for 72 h. Cell index curves showed pirfenidone dose-dependently inhibited the proliferation of fibroblasts. (**B**) Pirfenidone dose-dependently decreased BrdU incorporation (*n* = 3, **** *p* < 0.0001 for all groups) and (**C**) p-hIF cell numbers (*n* = 3, **** *p* < 0.0001 for all groups) after 72 h exposure. (**D**) Bright field images showing pirfenidone inhibited the proliferation of p-hIFs, while maintaining their spindle like morphology. (**E**) Stills of real-time cell imaging tracking the movement of individual p-hIFs after 0, 5, 10 and 15 h in the absence or presence of pirfenidone. (**F**) Quantification of p-hIF motility in each group shown in E. Motility was tracked every 5 min for 15 h in total. * *p* < 0.05; Data are shown in mean ± SEM. Scale bars = 100 μm.

**Figure 2:**
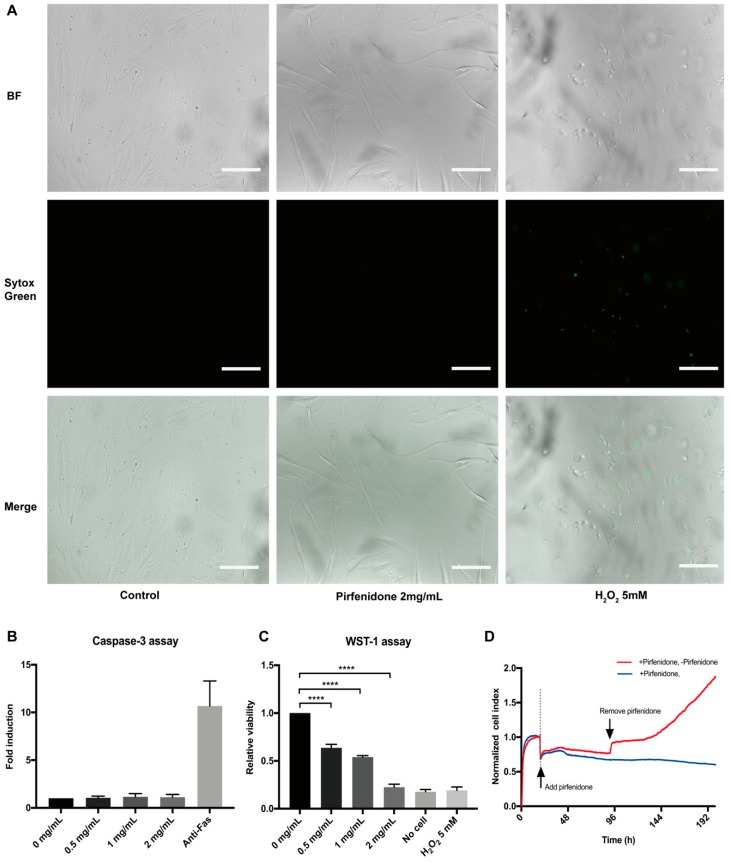
Pirfenidone does not induce cell death and reversibly suppresses the proliferation of human primary intestinal fibroblasts. (**A**) Bright field (top panels), Sytox Green nucleic staining (middle panels) and overlay images (bottom panels) of p-hIFs exposed for 72 h to 2 mg/mL pirfenidone or for 24 h to 5 mmol/L H_2_O_2_ (positive control for necrosis) showing that less than 1% of pirfenidone-exposed p-hIFs were necrotic (representative image of *n* = 3). (**B**) Pirfenidone did not induce caspase-3 activity compared to anti-Fas-exposed (1 ug/mL for 9 h) p-hIFs (positive control for apoptotic cell death) (*n* = 3). (**C**) Water Soluble Tetrazolium Salt-1 (WST-1) assay showed pirfenidone dose-dependently suppressed the total metabolic activity of p-hIFs as proxy for total cell number (**** *p* < 0.0001, *n* = 3). (**D**) Pirfenidone (2 mg/mL for 72 h)-induced inhibition of p-hIF proliferation was reversible even refreshing the cells with pirfenidone-free medium, as analyzed in real-time in the xCELLigence. Scale bars = 50 μm.

Primary hIFs (p-hIFs) were cultured in a real-time cell analysis (RTCA, xCELLigence) to analyze the effect of pirfenidone on cell proliferation. The p-hIFs were allowed to attach to the culture plate for 18 h, represented by stabilization of the Cell Index (CI). Next, the p-hIFs were treated with increasing concentrations of pirfenidone (0, 0.5, 1, and 2 mg/mL) for 72 h, which revealed that pirfenidone dose-dependently reduced the increase in CI (green, purple and blue lines) when compared to untreated control p-hIFs (red line in Figure 1A). In line, pirfenidone dose-dependently reduced BrdU incorporation (Figure 1B; all **** *p* < 0.0001 when compared to untreated control p-hIFs) and cell numbers (34%, 72%, and 97% at 0.5, 1.0, and 2.0 mg/mL, respectively; Figure 1C, all **** *p* < 0.0001). Video-assisted imaging of p-hIFs revealed that pirfenidone also suppressed the motility of individual p-hIF, albeit only significantly at the highest concentration of 2 mg/mL (Figure 1E,F). Pirfenidone treatment did not evidently affect the typical spindle-shaped cell morphology of p-hIFs (Figure 1D,E). Moreover, pirfenidone did not induce significant levels of necrotic p-hIF cell death (Figure 2A), nor did it induce caspase-3 activity as a measure of apoptotic cell death (Figure 2B). Still, 72 h pirfenidone treatment dose-dependently reduced the metabolic activity of p-hIFs, as quantified in WST-1 assays (Figure 2C; **** *p* < 0.0001 for at all tested concentrations). As pirfenidone did not appear cytotoxic for p-hIFs, we next analyzed whether p-hIFs proliferation is reversible after cessation of pirfenidone treatment. p-hIFs were pre-treated for 72 h with 2 mg/mL pirfenidone inhibiting cell proliferation and upon refreshing the medium without pirfenidone the p-hIFs regained normal proliferation rates after a lag-phase of approximately 48 h (Figure 2D). Notably, the cell index of pirfenidone pre-treated p-hIFs reached the same level after 96 h as compared to non-treated p-hIFs (see for reference Figure 1A).

### 3.2. Pirfenidone Suppresses Extracellular Matrix Protein (ECM) Expression in p-hIFs

**Figure 3:**
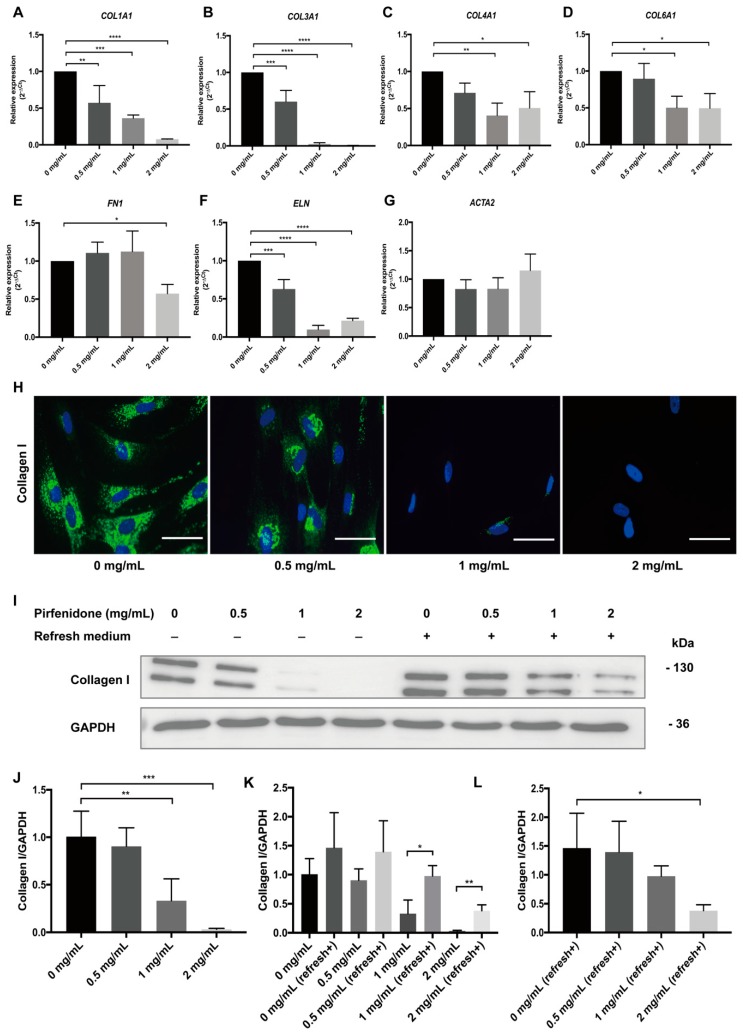
Pirfenidone inhibits Extracellular Matrix Protein (ECM) production. (**A**–**G**) Intestinal fibroblasts were treated with increasing (maximum 2 mg/mL) concentrations of pirfenidone for 72 h. Pirfenidone suppressed *COL1A1*, *COL3A1*, *COL4A1*, *COL6A1*, *FN1*, and *ELN* mRNA levels, but not *ACTA2* levels (* *p* < 0.05, *n* = 3). (**H**) Immunofluorescent images showing that pirfenidone dose-dependently suppressed intracellular levels of collagen I. (**I**) p-hIFs were exposed for 72 h to pirfenidone (Lanes 1–4) and subsequently cultured in pirfenidone-free medium for 96 h (Lanes 5–8). (**J**–**L**) Western blot analysis showed that pirfenidone concentration-dependently and reversibility suppressed collagen I protein levels in p-hIFs. The relative abundance of the tested proteins was normalized to that of Glyceraldehyde 3-phosphate dehydrogenase (GAPDH). ** *p* < 0.01, *** *p* < 0.001, **** *p* < 0.0001; Data are presented as mean ± SEM. Scale bars = 50 μm. Refresh+, refresh medium.

In order to determine whether pirfenidone also affects ECM production, p-hIFs were exposed to pirfenidone for 72 h and mRNA levels of genes encoding ECM proteins, as well as collagen I protein production were analyzed by RT-qPCR, IF, and Western blot analysis (Figure 3). Pirfenidone dose-dependently reduced *COL1A1*, *COL3A1*, *COL4A1*, *COL6A1*, *FN1* (encoding fibronectin 1) and *ELN* (encoding elastin) mRNA levels, while it did not affect *ACTA2* (encoding alpha-smooth muscle actin; α-SMA) expression (Figure 3A–G). Untreated p-hIFs contained high levels of (intracellular) collagen I, which was dose-dependently reduced by pirfenidone and was virtually absent after 72 h exposure with 2 mg/mL pirfenidone (Figure 3H), also when analyzed by Western blotting (Figure 3I,J). When 72 h-pirfenidone treated p-hIFs were exposed to normal medium again, collagen I protein reappeared (Figure 3K), but even after 96 h collagen I protein levels were still clearly lower compared to p-hIFs that were not pretreated with pirfenidone (Figure 3I,L).

### 3.3. Pirfenidone Reduces TGF-β1-Induced COL1A1 mRNA Expression and Collagen I Synthesis

**Figure 4:**
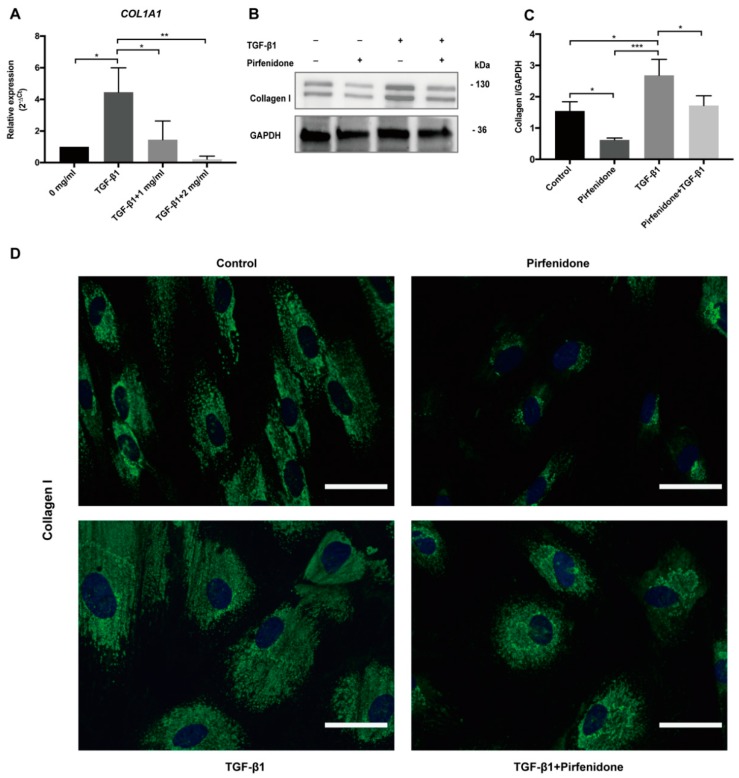
Pirfenidone suppresses basal and TGF-β1-induced collagen I expression in p-hIFs. (**A**) TGF-β1 (2.5 ng/mL) induces *COL1A1* mRNA levels. Pirfenidone dose-dependently suppresses TGF-β1-induced *COL1A1* mRNA levels (* *p* < 0.05, *n* = 3) which was accompanied with a reduction in collagen I protein expression levels (**B**,**C**). (* *p* < 0.05, *n* = 3; GAPDH is included as protein loading control) (**D**) Immunofluorescence microscopy revealed that TGF-β1 enhanced the cellular surface area positive for collagen I, which was reduced again by pirfenidone leading to a perinuclear location of collagen I. ** *p* < 0.01, *** *p* < 0.001; Data are presented as mean ± SEM. Scale bars = 50 μm.

Exposure of p-hIFs to TGF-β1 (2.5 ng/mL for 72 h) strongly increased *COL1A1* mRNA levels (>4-fold; Figure 4A), which was accompanied by increased collagen I protein levels (Figure 4B–D). Co-treatment with pirfenidone completely blocked the TGF-β1-induced expression of collagen 1, both at mRNA (pirfenidone at 1 and 2 mg/mL) and protein level (pirfenidone at 1 mg/mL) (Figure 4A–C). IF revealed that TGF-β1 induced collagen I protein accumulation in intestinal fibroblasts. Collagen I remained detectable in TGF-β1 + pirfenidone cotreated p-hIFs but it appeared to accumulate perinuclear, in comparison to more evenly distributed throughout the cellular cytoplasm in TGF-β1-only treated p-hIFs (Figure 4D).

### 3.4. Pirfenidone Inhibits TGF-β1-Mediated Phosphorylation of TGF-β1/mTOR/p70S6K Signaling Pathway in p-hIFs

**Figure 5:**
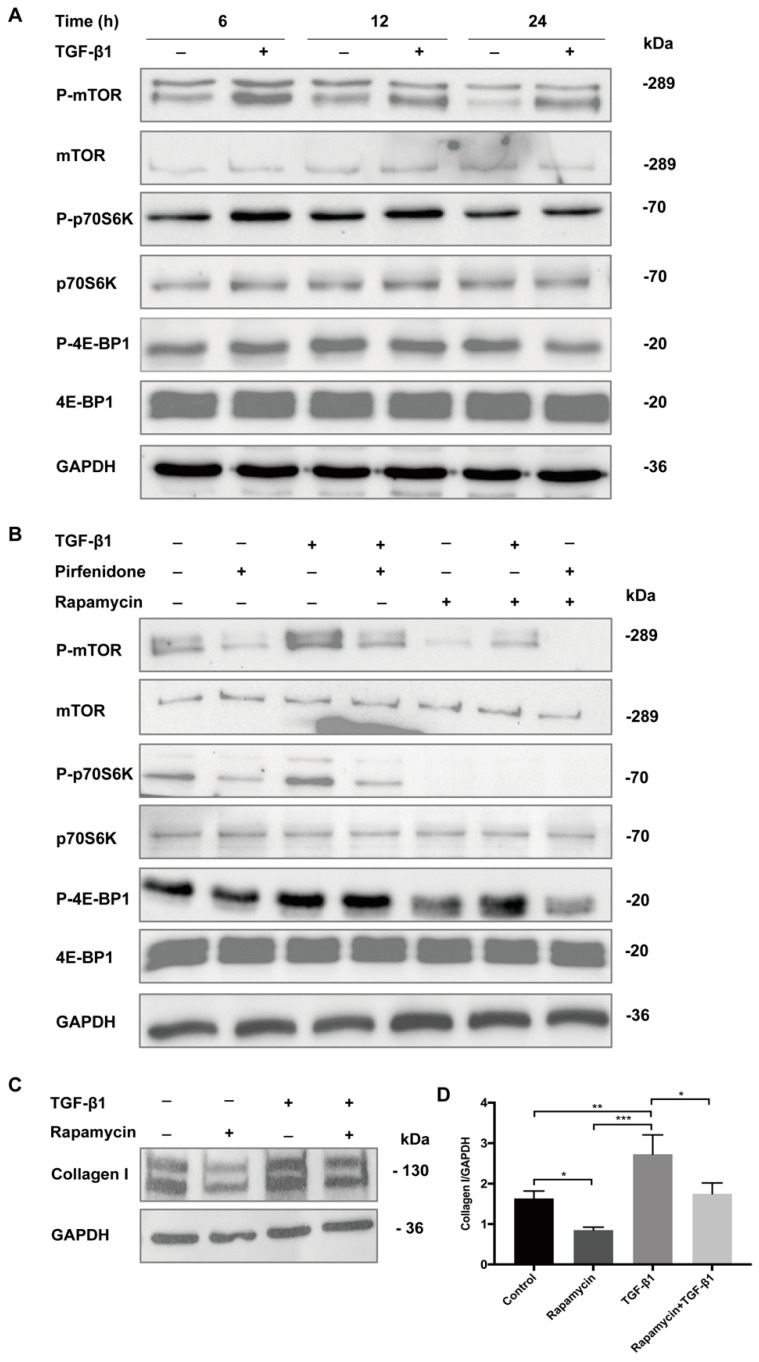
Pirfenidone suppresses the TGF-β1/mTOR/p70S6K signaling pathway. (**A**) p-hIFs were cultured for 6, 12, and 24 h in the absence or presence of TGF-β1 (2.5 ng/mL). Levels of total and phosphorylated mTOR, p70S6K, and 4E-BP1 were assessed by Western blot analysis. TGF-β1 enhanced the levels of p-mTOR (at 6 h) and p-p70S6K (at 6–12 h). (**B**) p-hIFs were exposed to TGF-β1 (2.5 ng/mL), pirfenidone (1 mg/mL), rapamycin (100 nmol/L), or combinations of those compounds for 6 h and analyzed by Western blotting. Pirfenidone and rapamycin inhibited basal and TGF-β1-induced phosphorylation of mTOR and p70S6K. (**C**,**D**) Rapamycin (100 nmol/L for 72 h) inhibited basal and TGF-β1-induced collagen I production in p-hIFs. * *p* < 0.05, ** *p* < 0.01, *** *p* < 0.001, *n* = 3; GAPDH is included as protein loading control.

In order to delineate the molecular signaling pathways that underlie the anti-proliferative/anti-fibrotic effects of pirfenidone, we analyzed its effect on Smad2/3, p38 MAPK and mTOR phosphorylation in p-hIFs in the presence and absence of TGF-β1. As shown in Supplementary Figure S1, both TGF-β1 and pirfenidone did not significantly affect phosphorylation of Smad2/3 and p38 MAPK in p-hIFs. Besides activation of the Smad-signaling [24,25,26,27,28,29], TGF-β1 may also signal via mTOR [30,31], the latter not being studied earlier in intestinal fibroblasts. Indeed, TGF-β1 enhanced mTOR phosphorylation (at 6 h) in p-hIFs, as well as downstream signaling factor p70S6K (at 6 and 12 h) (Figure 5A). In contrast, TGF-β1 did not affect the levels of phosphorylated 4E-BP1 at any of the analyzed time points (6, 12, and 24 h TGF-β1 exposure) (Figure 5A). Pirfenidone (1 mg/mL) strongly suppressed both basal and TGF-β1-induced phosphorylation of mTOR and p70S6K, while it hardly affected 4E-BP1 phosphorylation (Figure 5B). Similar effects were observed for rapamycin (100 nmol/L, Figure 5B), the classical mTOR signaling pathway inhibitor. In line, rapamycin (100 nmol/L) also suppressed basal and TGF-β1-induced expression of collagen I in p-hIFs (Figure 5C).

Taken together, our data show that pirfenidone suppressed proliferation of p-hIFs and collagen I production by p-hIFs. Pirfenidone inhibits TGF-β1-induced mTOR signaling that may aid in the inhibition of p-hIFs proliferation and collagen production.

## 4. Discussion

In this study, we show that pirfenidone dose-dependently and reversibly inhibits the proliferation and excessive production of ECM components, in particular collagen I. We show for the first time that pirfenidone suppresses basal and TGF-β1-induced mTOR signaling in p-hIFs, a pathway known to contribute to organ fibrosis. Thus, pirfenidone is a relevant drug to explore further for the treatment of intestinal fibrosis, a condition often associated with chronic intestinal diseases, such as IBD.

Intestinal inflammation triggers the activation of myofibroblasts that are central players in tissue repair, as they migrate into damaged tissue and control production and turnover of the extracellular matrix in support of all the other tissue-resident cell types. However, chronic inflammation leads to overactivation and proliferation of myofibroblasts that produce excessive amounts of ECM causing fibrosis [32,33]. Therefore, suppressing myofibroblast proliferation and/or ECM production is an important therapeutic target to control and prevent intestinal fibrosis. An earlier study analyzed p-hIFs from CD patients and found that pirfenidone inhibited cell proliferation, but did not affect collagen (I-V) production [34]. The p-hIFs were exposed to the same concentrations of pirfenidone as we did, however, only for 24 h. Moreover, only secreted collagen (in the medium) was quantified and no difference were found for untreated and pirfenidone-treated p-hIFs. Given the fact that pirfenidone did not affect collagen production in their study, they concluded that it may be of limited clinical value for treating intestinal fibrosis. We, however, found that pirfenidone dose-dependently suppresses *COL1A1*, *COL3A1*, *ELN* (all >90%) and *COL4A1*, *COL6A1* and *FN1* (>50%) mRNA levels, as well as collagen I protein production by p-hIFs, along with inhibiting cell proliferation. The key difference probably is that we treated the p-hIF for 72 h and the most pronounced effect of pirfenidone, also on cell proliferation, is observed only 24 h after the treatment started, especially at the lower concentrations of pirfenidone (Figure 1A). In addition, we show that TGF-β1-induced collagen I production is effectively suppressed by pirfenidone. Collagen production is a lengthy process, including intracellular and extracellular post-translation modifications (prolyl-hydroxylation), triple helix formation and processing of pro-peptides. It is therefore conceivable that effects of inhibiting this process may become evident only after 24 h. We confirm earlier results that the pirfenidone-induced inhibition of p-hIF proliferation is reversible and did not induce cell death. However, collagen I production was still significantly reduced 96 h after cessation of pirfenidone exposure, when compared to untreated p-hIFs. The prolonged effect of one treatment of pirfenidone on p-hIFs may allow intermittent dosing of this drug in patients and thereby reduce possible adverse side effects, when compared to daily administration. The fact that pirfenidone does not induce p-hIF cell death is also advantageous, as these cells play essential roles in wound healing and tissue repair [33,35]. The reversible effect of pirfenidone on cell proliferation makes it therefore flexible to be used between wound healing and anti-fibrosis processes.

**Figure 6:**
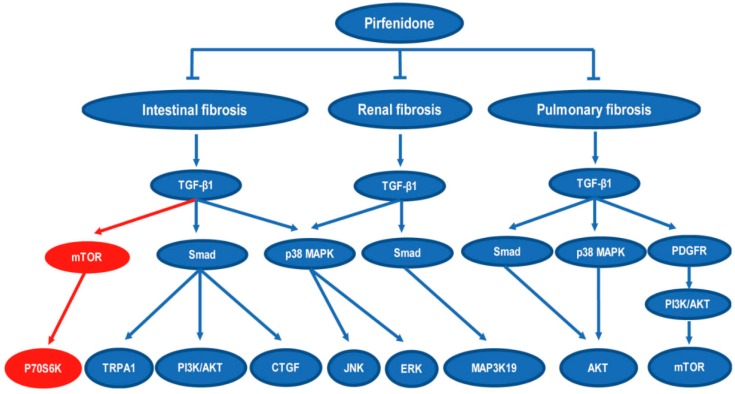
Overview of signaling pathways involved in the development of tissue fibrosis that are affected by pirfenidone. Previously reported pathways are presented in blue. The mTOR pathways studied in this work is presented in red.

Our results are largely in line with earlier observations using human intestinal fibroblast cell lines and rat and mouse primary intestinal fibroblasts. Pirfenidone was shown to suppress TGF-β1-induced *COL1A1* and *ACTA2* expression in a human intestinal myofibroblast cell lines either by activating the transient receptor potentialankyrin 1 channel [24], by inhibiting Smad/PI3K/AKT [27] signaling or the MAPK pathway [25]. Many studies have analyzed the effect of pirfenidone on tissue fibrosis in organs other than the intestine, and multiple pathways have been implicated with most focus on Smad-related signaling pathways (summarized in Figure 6) [18,24,25,26,27,28,29,30,36,37]. However, compared to the earlier studies using intestinal fibroblasts, we found that a 6 h exposure to TGF-β1 (2.5 ng/mL) did not significantly induce phosphorylation of Smad 2/3 or p38-MAPK in p-hIFs. This may be because we exposed the p-hIFs to lower concentrations TGF-β1 compared to the other studies (5-10 ng/mL; [24,25,26,27]). TGF-β1 induces Smad2/3 phosphorylation within minutes [24] and elevated levels are still detectable after 24–48 h exposure [25,26,27]. Still, the extent of Smad2/3 phosphorylation is time-dependent and the time-point we chose (6 h of TGF-β1 treatment) may not have been optimal. Sun et al. also examined the effect of pirfenidone on “untreated” human intestinal fibroblasts and found that this did not lower Smad 2/3 phosphorylation, which is in line with our data. Notably, phosphorylated Smad2/3 and p38 MAPK were detectable in control-cultured p-hIFs, which may indicate a higher basal activation state compared to CCD-Co18 [25], InMyoFib [24] and HUM-CELL-d022 [27] fibroblasts. A possible effect of pirfenidone on mTOR signaling has so far not been studied for intestinal fibrosis. We show that 2.5 ng/mL TGF-β1 quickly enhances mTOR phosphorylation, followed by downstream p70S6K phosphorylation. This suggests that the mTOR pathway was more sensitive to the stimulation of TGF-β1 than Smad 2/3 and p38-MAPK pathways in p-hIFs. Rapamycin-sensitive mTOR signaling plays an important role in regulating cell growth, cell proliferation, protein synthesis, transcription and autophagy [38]. p70S6K is one of the main key substrates of mTOR and promotes protein synthesis at the ribosome [39]. Pirfenidone inhibited both basal and TGF-β1-induced mTOR and p70S6K phosphorylation which may suppress collagen production. Indeed, treating p-hIFs with rapamycin, the prototypical inhibitor on mTOR [40] also suppressed both basal and TGF-β1-induced collagen I production in p-hIFs. This supports the relationship between mTOR signaling and collagen I production in p-hIFs and suggests that pirfenidone, at least in part, acts via mTOR to suppress ECM production and p-hIF proliferation.

It is important to note that besides anti-fibrotic activity, pirfenidone has also been shown to act as an anti-inflammatory and an anti-oxidant agent in IPF patients [41,42]. Here, we specifically focused on the potential anti-fibrotic action of p-hIFs, but the anti-inflammatory and anti-oxidant activities of pirfenidone may synergize in its therapeutic effect in patients with IBD. One important aspect of pirfenidone has to considered, as it has been reported to cause some gastrointestinal side effects in IPF patients, including nausea, vomiting, dyspepsia and diarrhea [43,44]. Current therapies for IBD, however, are also pursuing intestine-specific delivery of drugs. The ColoPulse technology is such a novel approach which allows controlled release of drugs at the terminal ileum and colon [45]. Systemic and gastrointestinal side effects of pirfenidone may be minimized using such approach, concomitant with a higher drug-efficacy of pirfenidone on intestinal inflammation and fibrosis [46].

In this study, we made use of p-hIFs from healthy tissue, which were exposed to TGF-β1, being a “master cytokine” in the development of tissue fibrosis. It remains to be determined to what level this exactly resembles the phenotype of fibroblasts residing in stenotic intestinal tissue in vivo. In addition, future studies need to establish the interindividual, as well as location-specific differences between intestinal fibroblasts and their value as in vitro models to study intestinal fibrosis.

In summary, we show that pirfenidone suppresses the proliferation and collagen production of primary intestinal fibroblasts, and it does so, at least in part, by inhibiting mTOR/p70S6K signaling. This study suggests that pirfenidone might be a potential new therapeutic drug for the treatment of intestinal fibrosis.

## Supplementary Materials

The following are available online at https://www.mdpi.com/2073-4409/9/3/775/s1, Figure S1: Pirfenidone does not suppress Smad2/3 and p38 MAPK phosphorylation in p-hIFs, Table S1: TaqMan primers and probes for Real-time Quantitative PCR analysis, Table S2: SYBR green primer sequences used for Real-time Quantitative PCR Analysis, Table S3: Antibodies catalog numbers and dilutions.
