# Supplementary material for: Pirfenidone Inhibits Cell Proliferation and Collagen I Production of Primary Human Intestinal Fibroblasts
Source: Cells. 2020 Mar 22;9(3):775. doi: 10.3390/cells9030775 (PMC7140656; doi:10.3390/cells9030775)
Supplement: Supplementary file 1 [file cells-09-00775-s001.pdf]

cells MDPI

Supplementary tables:

Table 1. TaqMan primers and probes for Real-time Quantitative PCR analysis.

Gene	TaqMan primers and probes
18S	Forward: CGGCTACCACATCCAAGGA
	Reverse: CCAATTACAGGGCCTCGAAA
	Probe: CGCGCAAATTACCCACTCCCGA
COL1A1	Forward: GGCCCAGAAGAACTGGTACATC
	Reverse: CCGCCATACTCGAACTGGAA
	Probes: CCCCAAGGACAAGAGGCATGTCTG
ACTA2	Forward: GGGACGACATGGAAAAGATCTG
	Reverse: CAGGGTGGGATGCTCTTCA
	Probe: CACTCTTTCTACAATGAGCTTCGTGTTGCCC

Table 2. SYBR green primer sequences used for Real-time Quantitative PCR Analysis.

Gene	SYBR green primers
COL3A1	Forward: CTGGACCCCAGGGTCTTC
	Reverse: CATCTGATCCAGGGTTTCCA
COL4A1	Forward: AGGAGAGAAGGGCGCTGT
	Reverse: TCCAGGTAAGCCGTCAACA
COL6A1	Forward: GAAGAGAAGGCCCCGTTG
	Reverse: CGGTAGCCTTTAGGTCCGATA
FN1	Forward: CTGGCCGAAAATACATTGTAAA
	Reverse: CCACAGTCGGGTCAGGAG
ELN	Forward: CGGGAGTAGTTGGTGTCCC
	Reverse: AGCTGCTTCTGGTGACACAA

Table 3. Antibodies catalog numbers and dilutions.

Antibody	Cat no. (dilution)	Company
GAPDH	CB1001 (1:1000)	Calbiochem
Phospho-mTOR	2971 (1:1000)	Cell Signaling
mTOR	2983T (1:1000)	Cell Signaling
Phospho-p70S6K Thr389	9205 (1:1000)	Cell Signaling
p70S6K	9202S (1:1000)	Cell Signaling
Phospho-4E-BP1 Ser65	9451 (1:1000)	Cell Signaling
4E-BP1	9644T (1:1000)	Cell Signaling
Phosho-Smad2 (S465/467)/3 (S423/425)	8828 (1:1000)	Cell Signaling
Smad2/3	3120 (1:1000)	Cell Signaling
Phosho-p38 MAPK	9211 (1:1000)	Cell Signaling
p38 MAPK	9212 (1:1000)	Cell Signaling
Polyclonal Rabbit Anti-Mouse Immunoglobulins	P0260 (1:2000)	DAKO
Polyclonal Rabbit Anti-Goat Immunoglobulins	P0160 (1:2000)	DAKO
Polyclonal Goat Anti-Rabbit Immunoglobulins	P0448 (1:2000)	DAKO

Supplementary Figure:

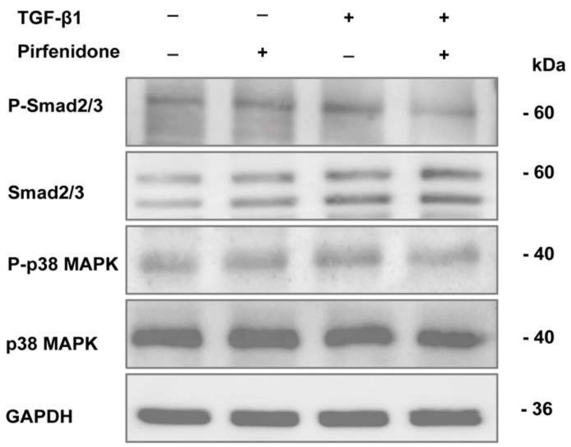

Figure 1. Pirfenidone does not suppress Smad2/3 and p38 MAPK phosphorylation in p-hIFs. (**A**) p-hIFs were exposed to pirfenidone (1 mg/ml) and/or, TGF-β1 (2.5 ng/ml) for 6 h. Total and phosphorylated Smad2/3 and p38 MAPK were assessed by Western blot analysis. Compared with the control group, TGF-β1 or pirfenidone did not pronouncedly change levels of phosphorylated Smad2/3 or p38 MAPK in p-hIFs. GAPDH is included as protein loading control.

Supplementary References for Figure 6:

1. Kurahara L.H.; Hiraishi K.; Hu Y.; et al. Activation of Myofibroblast TRPA1 by Steroids and Pirfenidone Ameliorates Fibrosis in Experimental Crohn's Disease. *Cell. Mol. Gastroenterol. Hepatol*. **2018**, *5*, 299–318.
2. Sun Y.; Zhang Y.; Chi P. Pirfenidone suppresses TGFbeta1induced human intestinal fibroblasts activities by regulating proliferation and apoptosis via the inhibition of the Smad and PI3K/AKT signaling pathway. *Mol. Med. Rep*. **2018**;*18*, 3907–3913.
3. Li G.; Ren J.; Hu Q.; et al. Oral pirfenidone protects against fibrosis by inhibiting fibroblast proliferation and TGF-beta signaling in a murine colitis model. *Biochem. Pharmacol*. **2016**;*117*, 57–67.
4. Sun Y.W.; Zhang Y.Y.; Ke X.J.; Wu X.J.; Chen Z.F.; Chi P. Pirfenidone prevents radiation-induced intestinal fibrosis in rats by inhibiting fibroblast proliferation and differentiation and suppressing the TGF-beta1/Smad/CTGF signaling pathway. Eur. J. Pharmacol. **2018**, *822*, 199–206.
5. Li Z.; Liu X.; Wang B.; et al. Pirfenidone suppresses MAPK signalling pathway to reverse epithelial-mesenchymal transition and renal fibrosis. *Nephrology* **2017**;*22,* 589–597.
6. Ji X.; Naito Y.; Weng H.; et al. Renoprotective mechanisms of pirfenidone in hypertension-induced renal injury: Through anti-fibrotic and anti-oxidative stress pathways. Biomed. Res. **2013**, *34*, 309–319.
7. Conte E.; Gili E.; Fagone E.; Fruciano M.; Iemmolo M.; Vancheri C. Effect of pirfenidone on proliferation, TGF-beta-induced myofibroblast differentiation and fibrogenic activity of primary human lung fibroblasts. Eur. J. Pharm. Sci. **2014**, *58*, 13–19.
8. Boehme S.A.; Franz-Bacon K.; DiTirro D.N.; Ly T.W.; Bacon K.B. MAP3K19 Is a Novel Regulator of TGF-beta Signaling That Impacts Bleomycin-Induced Lung Injury and Pulmonary Fibrosis. *PloS One* **2016**, *11*, doi:10.1371/journal.pone.0154874
9. Kurita Y.; Araya J.; Minagawa S.; et al. Pirfenidone inhibits myofibroblast differentiation and lung fibrosis development during insufficient mitophagy. *Respir. Res*. **2017**, *18*, 114.
10. Molina-Molina M.; Machahua-Huamani C.; Vicens-Zygmunt V.; et al. Anti-fibrotic effects of pirfenidone and rapamycin in primary IPF fibroblasts and human alveolar epithelial cells. *BMC Pulm. Med.* **2018**, *18*, 63.
